# Pre-operative mechanical bowel preparation and prophylactic oral antibiotics for pediatric patients undergoing elective colorectal surgery: a protocol for a randomized controlled feasibility trial

**DOI:** 10.1186/s40814-024-01476-6

**Published:** 2024-05-25

**Authors:** Daniel Briatico, Helene Flageole, Noora Al-Shahwani, Forough Farrokhyar, Lisa VanHouwelingen

**Affiliations:** 1 McMaster Pediatric Surgery Research Collaborative, McMaster University, Hamilton, ON Canada; 2 Division of Pediatric General Surgery, Department of Surgery, McMaster University, Hamilton, ON Canada; 3 Department of Surgery, Sidra Medical and Research Center, Doha, Qatar; 4 Department of Health Research Methods, Evidence & Impact, Department of Surgery, McMaster University, Hamilton, ON Canada

**Keywords:** Pediatric, Colorectal surgery, Surgical site infection, Antibiotics, Mechanical bowel preparation

## Abstract

**Background:**

Infections after elective colorectal surgery remain a significant burden for patients and the healthcare system. Adult studies suggest that the combination of oral antibiotics and mechanical bowel preparation is effective at reducing infections after colorectal surgery. In children, there is limited evidence for either of these practices and the utility of combining oral antibiotics with mechanical bowel preparation remains uncertain.

**Methods:**

This study aims to determine the feasibility of conducting a randomized controlled trial assessing the efficacy of oral antibiotics, with or without mechanical bowel preparation, in reducing the rates of post-operative infection in pediatric colorectal surgery. Participants aged 3 months to 18 years undergoing elective colorectal surgery will be randomized pre-operatively to one of three trial arms: (1) oral antibiotics; (2) oral antibiotics and mechanical bowel preparation; or (3) standard care. Twelve patients will be included in each trial arm. Feasibility outcomes of interest include the rate of participant recruitment, post-randomization exclusions, protocol deviations, adverse events, and missed follow-up appointments. Secondary outcomes include the rate of post-operative surgical site infections, length of hospital stay, time to full enteral feeds, reoperation, readmission, and complications.

**Discussion:**

If the results of this trial prove feasible, a multi-center trial will be completed with sufficient power to evaluate the optimal pre-operative bowel preperation for pediatric patients undergoing elective colorectal surgery.

**Trial registration:**

ClinicalTrials.gov: NCT03593252.

## Background

Colorectal surgery is associated with high rates of surgical site infections (SSI) in both adult and pediatric populations, occurring in up to 25% of all cases [1]. The American College of Surgeons (ACS) National Surgical Quality Improvement Program (NSQIP) pediatric data shows that colorectal procedures account for 2.5% of all pediatric surgical cases but contribute to 7.1% of the SSI burden [2]. Many of the Canadian centers that currently participate in the NSQIP pediatric program, including our hospital, have higher than expected rates of SSIs in elective colorectal cases. This serves as a great impetus to find the optimal SSI reduction strategy for these patients.

Two pre-operative techniques have been proposed as potential methods of reducing infection in colorectal surgery, oral antibiotics (OA) and mechanical bowel preparation (MBP), which aims to cleanse the large bowel of its fecal contents. The evidence is weak for either of these techniques in the pediatric population, and practice patterns remain variable and largely dependent on surgeon preference. A survey conducted by the American Pediatric Surgical Association (APSA) in 2015 showed that the most common approach taken among pediatric surgeons for all intestinal procedures was MBP alone (31.1%), followed by dietary modifications (26.8%), and OA combined with MBP (19.6%) [2]. The current approach at McMaster Children’s Hospital in pediatric patients undergoing a colorectal procedure is intravenous antibiotics with good coverage against gut flora given on induction of anesthesia, without any bowel preparation.

MBP alone has fallen out of favor due to the paucity of beneficial evidence (i.e., clearing the bowel of fecal content does not necessarily reduce the quantity of bacteria in the intestinal mucosa) and the potential increased risk of post-operative complications [3–6]. A Cochrane Review, encompassing 18 randomized controlled trials (RCTs), showed no difference in the rate of wound infection or anastomotic leak in colorectal procedures between patients who underwent MBP and those who did not [4]. More recently, two systematic reviews and meta-analyses support those findings [5, 6]. Lok et al. identified two RCTs and four retrospective cohort studies of patients 0–21 years of age undergoing elective colorectal surgery and assessed the effect of pre-operative MBP on the incidence of post-operative complications including anastomotic leak, wound infection, and intra-abdominal infection [5]. Overall, MBP before colorectal surgery did not decrease the incidence of post-operative infectious outcomes [5]. Similarly, Zwart et al. conducted a meta-analysis on two RCTs and four retrospective comparative studies and found that among patients aged 0–21 years undergoing colorectal surgery, the risk of developing a post-operative infection was 10.1% in patients who received MBP compared to 9.1% in patients who did not receive MBP, resulting in a non-significant risk difference of − 0.03% (95% CI − 0.09 to 0.03%) [6].

With regard to OA alone versus no preparation, the adult literature shows promising results in favor of the use of OA. In a Cochrane Review on antimicrobial prophylaxis in colorectal surgery (*n* = 260 RCTs), the addition of OA to intravenous antibiotics was found to reduce surgical wound infection (RR = 0.56, 95% CI 0.43 to 0.74) [7].

Evaluating a combination approach, a recent network meta-analysis by Toh et al. assessed 8458 adult patients (*n* = 38 RCTs), comparing four bowel preparation groups: (1) OA with MBP; (2) OA only; (3) MBP only; and (4) no preparation [8]. The primary outcome was the total rate of incisional and organ space SSIs. Results showed that MBP with OA was associated with the lowest risk of SSI [8]. The use of OA without MBP was not associated with a significant reduction in SSI [8]. Lastly, there was no difference between MBP only and no preparation [8].

Contrary to the extensive body of research available in adults, there is a noticable gap in studies examining pre-operative bowel preparation for colorectal surgery in children. Moreover, available studies tend to be retrospective in nature with smaller sample sizes. A multi-center, retrospective study by Serrurier et al. assessed outcomes in 272 children who underwent colostomy takedown and found higher rates of wound infection (14% vs 6%, *p* = 0.04) and a longer length of hospital stay among those who received MBP versus no preparation [9]. Another study by Breckler et al. comparing MBP versus OA + MBP in 118 children undergoing colostomy closure for imperforate anus found no difference in SSI rate (13% vs 17%, *p* = 0.64) [10]. In a more recent retrospective cohort study using administrative data, Ares et al. analyzed 1581 pediatric patients undergoing elective colorectal procedures and found post-operative complications to be the highest in the group who received no pre-operative preparation, when compared to those receiving combination preparation (OA + MBP), and MBP alone (23.3%, 15.9%, and 14.2%, respectively; *p* = 0.002) [11]. Interestingly, their findings suggest the addition of OA may be deleterious [11]. Evidence to support the sole use of oral antibiotics opposed to in combination with MBP is lacking, particularly in the pediatric literature.

Due to the existing variability in surgeon preference and the lack of sufficient prospective evidence for either combination OA + MBP or OA alone, we believe that there is a need for a well-designed clinical trial. Before conducting a fully powered multicentre trial, this feasibility study will provide important and pragmatic information on resource requirements, logistics, protocol testing, intervention adherence, and risk assessment.

## Methods

### Study design and objective

**Fig. 1:**
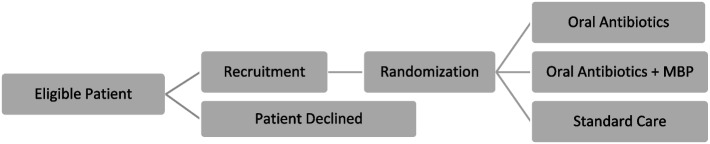
Trial design

This trial design is a parallel, randomized controlled feasibility trial. The trial design is outlined in Fig. 1.

The primary objective of this trial is to determine the feasibility of conducting a RCT to assess the efficacy of OA, with or without the use of MBP, in reducing the rate of post-operative infections in pediatric patients undergoing elective colorectal surgery and to validate key assumptions for a large-scale superiority trial.

#### Study setting

This study will be conducted at McMaster Children’s Hospital, a pediatric tertiary care teaching hospital located in Hamilton, Ontario, Canada. Pediatric patients undergoing elective colorectal surgery will be screened and recruited from Pediatric General Surgery outpatient clinic.

#### Inclusion criteria

The specific inclusion criteria for this study are the following:

1. Pediatric patients aged 3 months to 18 years being treated by the Pediatric General Surgery service at McMaster Children’s Hospital
2. Undergoing elective colorectal surgery
3. Parents or legal guardians able to give free and informed consent
4. For females of childbearing age, a negative pregnancy test

These criteria reflect the characteristics of children/adolescents undergoing elective colorectal procedures for which the results will be generalizable. Elective colorectal procedures eligible for this study include (but not limited to) the following:

1. Colostomy closure
2. Large bowel resection with creation of ostomy or anastomosis/pull-through
3. Trans-anal pull-through
4. Anorectoplasty

#### Exclusion criteria

The specific exclusion criteria for this study are the following:

1. Non-elective surgery
2. Procedures that would not require MBP
  Colorectal resection with an existing proximal diverting ostomy
  Closure of small bowel ostomy
3. Mechanical bowel obstruction
4. Known hypersensitivity to laxatives (pico-salax and sennaquil) or OA (neomycin and metronidazole)
5. Contraindication to OA 
6. Patients on long-term antibiotics (1 month or more) directly before surgery
7. Congestive heart failure
8. Renal insufficiency (eGFR < 60 ml/min per 1.73 m^2^; Schwartz equation [12])
9. Cockayne syndrome
10. Pregnant and/or nursing
11. Other medical conditions precluding the use of either OA or MBP 
12. Co-enrolment in another intervention trial

#### Interventions

Patients will be randomized to receive either OA + MBP, OA alone, or no preparation (standard of care). If the patient/parents choose not to participate in the trial, the preparation regimen will be at the discretion of the treating surgeon. The trial interventions are detailed in Table 1.

**Table 1:** Trial interventions

**Standard care** (all groups)	**Oral antibiotics**	**Mechanical bowel preparation**
• Care according to institutional SSI reduction bundle	Oral non-absorbable antibiotics • Neomycin (15 mg/kg/dose, max of 1 g) To be given the day before surgery at 3 pm, 6 pm, and 9 pm	Two days before surgery No dietary restrictions
		• < 1 year: 5 mL Sennaquil at 4 pm • 1–5 years: 10 mL Sennaquil at 4 pm • > 6 years: 15 mL Sennaquil at 4 pm
	Oral absorbable antibiotics • Metronidazole (15 mg/kg/dose, max of 500 mg) To be given the day before surgery at 3 pm and 9 pm	The day before surgery Light breakfast then clear fluid diet
		• < 1 year: 5 mL Sennaquil at 4 pm • 1–5 years: ¼ sachet Pico-Salax at noon and 4 pm • 6–12 years: ½ sachet Pico-Salax at noon and 4 pm • > 12 years: 1 sachet of Pico-Salax at noon and 4 pm
		The day of surgery
		Care according to standard of care protocol

#### Standard of care

All study patients, regardless of group allocation, will receive institutional standard of care, including utilization of a peri-operative SSI reduction bundle. The components of this bundle (which all patients will be treated according to) are the following:

1. Pre-operative chlorhexidine bath at home the night before surgery
2. Nil per os (NPO) protocol in line with anesthesia requirements
  Solids until 8 h before surgery
  Formula until 6 h before surgery
  Breast milk until 4 h before surgery or clear liquids until 2 h before
3. Avoidance of hair shaving before or in the operating room
4. Use of an alcohol-based skin preparation solution such as chlorhexidine, except for mucosal surfaces (e.g., stoma)
5. On mucosal skin surfaces, use of proviodine solution
6. Antibiotics given on induction and 1 h or less before incision
  Cefazolin (30 mg/kg, max 2 g) or metronidazole (15 mg/kg, max 500 mg)
  In case of allergy to penicillin or cephalosporins, ciprofloxacin (10 mg/kg, max 400 mg/dose) or gentamicin (2.5 mg/kg)
7. Maintenance of normothermia throughout surgery
8. Skin closure with clean instruments
  The surgery team (surgeons and scrub nurses) change their gowns and gloves and move to a new clean set of instruments at the time of skin closure to minimize contamination
9. Occlusive dressing applied for 48 h post-operatively

#### Participant timeline

The duration of the treatment period will vary depending on which treatment arm the patient is randomized to. Patients in the OA group will begin treatment 1 day before surgery. Those allocated to the OA + MBP group will begin the study treatments 2 days prior to surgery. All study treatments will be completed prior to surgery commencing.

#### Outcomes

##### Primary outcome measures

The primary outcome measures for this trial will assess the feasibility of conducting a full RCT. The outcomes of interest are the participant recruitment rate (percentage of eligible patients enrolled), the rate of post-randomization exclusions, the number of protocol deviations (compliance to pre-operative bowel preparation regimen), adverse events related to the use of MBP and OA, and missed follow-up appointments.

##### Secondary outcome measures 

The secondary outcomes of interest for this study are the rate of SSIs (both superficial and deep), length of hospital stay, time to full enteral feeds, re-operation (within 30 days of initial surgery), readmission (within 30 days of initial discharge), and complications.

#### Sample size

As this is a feasibility study focused predominantly on the research process [13, 14], a formal sample size calculation was not conducted. To assess whether the study’s procedures are practical and to estimate parameters like recruitment rate, treatment adherence, and variability of outcomes, a sample size of 12 per group was selected [15].

#### Recruitment

At this institution, it is estimated that approximately 50–60 pediatric patients undergo elective colorectal surgery each year. Based on a conservative recruitment rate of 60% (i.e., 2–3 patients per month), this trial will take approximately 2 years to complete.

Patients who meet the eligibility criteria will be identified by the treating staff surgeon through the Pediatric General Surgery outpatient clinic. Recruitment will begin once ethics approval is received from the Hamilton Integrated Research Ethics Board (HiREB) and Health Canada.

#### Consent or assent

The Research Coordinator will be responsible for obtaining informed consent/assent from each patient. The consent conversation will occur only after a clinical staff member within the patient’s circle of care obtains permission for the Research Coordinator to approach. Each family will receive a Letter of Information that summarizes the trial, their involvement, and potential risks. The Research Coordinator will review all this information with them, in detail as per the Tri-Council Policy Statement (TCPS-2) guidelines. The patient and/or family will also be given an opportunity to ask questions. Once informed consent is obtained, the patient will be enrolled in the study, and data collection will begin.

#### Randomization and sequence generation

Patients will be randomized to treatment groups using a computer-generated randomization list. This will consist of random blocks of multiple sizes (3, 6, 9), created by a senior biostatistician. Patients will be randomized according to a 1:1:1 parallel allocation, with an equal chance of being allocated to OA, OA + MBP, or standard care alone.

#### Concealment mechanism

The randomization scheme will be stored in Research Electronic Data Capture (REDCap), an online database housed at McMaster University [16, 17]. Patients will be randomized by the Research Coordinator, ensuring allocation concealment from study investigators. The Research Coordinator will then deliver the randomization number to the inpatient pharmacy, where the medication will be prepared and dispensed by a trained Research Pharmacist. The Research Pharmacist will possess a linkage key to determine which study arm the patient is randomized to, based on the randomization number provided by the Research Coordinator. The assigned treatments will be crosschecked against the master linkage key at the end of the study.

#### Implementation

Patients will be randomized following identification, assessment of study eligibility, and acquisition of patient/parental consent. Randomization will take place 2 weeks before surgery, 1 day before their pre-operative anesthesia consultation. The Research Coordinator will provide the Research Pharmacist with the randomization code prior to their pre-operative appointment. The Research Coordinator will pick up the study medication from the pharmacy and meet with the patient. At this time, they will provide them with the necessary medication and study materials. They will explain how to administer the medication and how to complete the stool diary. They will also answer any trial-related questions. Patients randomized to the standard care trial arm will still be required to complete the stool diary. In circumstances where patients are unable to travel to the hospital to pick up the study medication, the Research Pharmacist will ship medications to them, and all education will be conducted virtually.

#### Blinding

The Research Pharmacist and the Research Coordinator will not be blinded to treatment allocation. Due to the nature of MBP, the patient will be aware of which study arm they have been randomized to. Therefore, the patient will not be blinded.

Patient records will mention that they are part of this study and will include the study number, but the actual medications received (i.e., group allocation) will not be mentioned. The surgical team operating on the patient will be blinded to treatment allocation. The Clinical Outcome Assessor, who will conduct the inpatient post-operative assessments and outpatient follow-up assessment, will also be blinded. The statistician analyzing the data will have a de-identified dataset and will be blinded to group allocation to ensure unbiased analysis. For the purposes of blinding the data analyst, data from the stool diary will be withheld until analysis for all other outcomes is completed.

#### Follow-up

Follow-ups will be completed daily by a Clinical Outcome Assessor while patients are admitted in hospital. There will also be one outpatient follow-up appointment in the Pediatric General Surgery outpatient clinic approximately 2 weeks after surgery. This follow-up visit will coincide with the patient’s regularly scheduled follow-up clinic visit. If the patient is unable to attend the appointment or fails to show up, the follow-up will be conducted by telephone. Visits to the emergency department and readmissions to the hospital between surgery and follow-up will also be recorded. A second follow-up will be completed by the Research Coordinator via telephone 30 days post-discharge.

The family will also be instructed to notify the Research Coordinator in the event that there were complications experienced and managed at another institution or by a family doctor or pediatrician.

#### Loss to follow-up

Full study follow-up will be considered complete 30 days post-discharge. A patient may be discontinued from the study at any time if the family, parent, surgeon, or investigators strongly believe it is not in the patient’s best interest to continue participation. Because all study interventions occur pre-operatively and study follow-up does not differ significantly from the standard follow-up protocol at this institution, the study drop-out rate is anticipated to be low.

#### Data collection

Data collection will be completed by a trained Research Coordinator and Clinical Outcome Assessor. The data collection responsibilities of each are outlined below.

#### Research Coordinator

The Research Coordinator will be responsible for data collection beginning on the day of surgery. They will meet parents in the waiting area and will collect patient demographics, diagnosis, and procedure-related information. Compliance to pre-operative bowel preparation will be assessed using the stool diary. This diary will contain spaces to record the medication doses and the times of administration. The character of the stool will be assessed by parents using a Bristol Stool Chart. Parents will be instructed to return the stool diary to the Research Coordinator on the morning of surgery. The recruitment rate (percentage of eligible patients enrolled), rate of post-randomization exclusions, number of protocol deviations, adverse events, and the number of missed follow-up appointments will also be collected by the Research Coordinator. Patients/parents will be asked to contact the Research Coordinator if they suspect a post-operative infection, who will then refer them to the Clinical Outcome Assessor to confirm whether an infection is present. Finally, the Research Coordinator will contact the patient/parents via telephone 30 days post-discharge and will review the patient’s electronic medical record for any emergency visits during that period.

#### Clinical Outcome Assessor

The Clinical Outcome Assessor will be responsible for collecting data on post-operative infectious complications (superficial and deep SSIs). The SSI will be classified based on the Centres for Disease Control and Prevention definitions [18]. The Clinical Outcome Assessor will be a trained healthcare worker (attending surgeon, surgical fellow, or nurse practitioner) with experience in recognizing infectious complications. They will be responsible for daily patient follow-up in the immediate post-operative period while the patient is admitted to the hospital. They will also conduct the assessment during the follow-up clinic visit after hospital discharge. If a post-operative complication occurs, the mode of treatment will be recorded. If a bacterial culture was obtained, the organism cultured, antibiotics therapy, and duration of antibiotics will be recorded.

#### Statistical analysis

Demographics will be reported as counts with percentages for categorical variables and means with standard deviations for continuous variables. Feasibility outcome measures (e.g., recruitment rate, post-randomization exclusions, protocol deviations, and adverse events) will be reported as proportions. Secondary outcome measures, such as surgical site infections, re-operation, and complications will be reported as proportions. Length of hospital stay and time to full enteric feeds will be reported as means with standard deviation. In cases where continuous data does not follow a normal distribution, medians with interquartile ranges will be used. The SSI risk ratio and risk difference with 95% CIs will be calculated to estimate the minimum clinically important difference based on clinical data. All data will be analyzed with an intention-to-treat approach using IBM SPSS Statistics Version 25.

#### Data management

All data for this study will be entered into REDCap, a secure, online database designed specifically for the purposes of this trial. All data will be de-identified by substituting patient-specific identifiers with a coded study identification number, such as S102, to ensure anonymity and protect privacy. Any study files containing patient identifiers, including the master list linking participant medical record number to their study identification number, will be stored in password-protected Excel files on the secure drive for the Department of Surgery at McMaster University. This drive is protected by the McMaster University Firewall, and only research staff directly involved in the conduct of this trial will have access to these documents. Paper study forms, including signed consent forms, will be kept in a locked filing cabinet in the secure office space for the Department of Surgery. Only research staff directly involved in the conduct of this trial will have access to this cabinet.

Data checks will be completed throughout the trial to ensure the accuracy of all data entered into the REDCap database. These checks will be completed after subjects 4, 12, and 20, with additional checks done at random throughout the study. All study data will be kept for 15 years in compliance with Health Canada requirements and then destroyed.

#### Data monitoring

This study will be conducted at McMaster Children’s Hospital in partnership with the McMaster Pediatric Surgery Research Collaborative (MPSRC). The Principal Investigator and Research Coordinator will oversee the recruitment of patients, training of research personnel, data collection, and adherence to protocol. They will liaise with all study team members including physicians, residents, and the nurse practitioner to ensure protocol compliance.

This study will also employ a Steering Committee. The Steering Committee members will be responsible for reviewing all protocol deviations, recruitment challenges, and any other significant issues that arise. The Steering Committee will meet every 3 months and will include representatives from Pediatric General Surgery, Infectious Disease, and Biostatistics.

#### Data Safety Monitoring Board

This study will utilize a Data Safety and Monitoring Board (DSMB). The DSMB will be a collaboration of three expert healthcare professionals independent of the Steering Committee and Study Investigational Team who will monitor patient safety during the conduct of the trial. Their role is to review all adverse event (AE) and serious adverse event (SAE) reports from the trial and submit a summary to the Steering Committee and Research Ethics Board. Based on these reports, the DSMB may recommend that the trial be discontinued for safety concerns; however, DSMB members cannot stop the trial for benefit. The DSMB will meet after the first five participants have completed their 30-day post-operative observation. The DSMB will then meet every 6 months thereafter. As needed, the DSMB will convene within 15 days following the notification of any potentially study-related SAE that is non-life-threatening or non-fatal. Additionally, the DSMB will convene within 48 h upon receiving reports of any potentially study-related life-threatening or fatal SAEs. This may be conducted via email or teleconference. To limit bias, DSMB members will be blinded to participant study group allocation.

#### Adverse events

In this study, an AE will be defined as any untoward medical occurrence following administration of a pharmaceutical product that does not necessarily have a causal relationship with this treatment [19]. An AE may be any unfavorable and unintended sign (including an abnormal laboratory finding), symptoms, or disease temporally associated with the use of a medicinal (investigational) product, whether or not related to the medicinal (investigational) product [19]. A SAE will be defined as any untoward medical occurrence that at any dose results in death, is life-threatening, requires inpatient hospitalization or prolongation of existing hospitalization, results in persistent or significant disability/incapacity, or is a congenital anomaly/birth defect [19]. The reporting of all AEs and SAEs will adhere to the guidelines set forth by the HiREB and Health Canada.

In the case of an AE, a qualified investigator (i.e., Principal Investigator or Co-Investigator) will assess the severity of the event and the outcome will be documented in the patient’s case report form, as well as in their electronic medical record. If an unexpected SAE occurs, the Principal Investigator will inform the DSMB within 48 h and notify the HiREB and Health Canada within 7 days of becoming aware of the occurrence.

#### Protocol amendments

Modification of the study protocol may affect the conduct of the study. This includes changes to the study design, investigators, objectives, sample size, procedures, and data collection forms.

Amendments will be formally submitted to HiREB and Health Canada after thorough discussion and consensus amongst the Principal Investigator, Co-Investigators, and Research Coordinator. Patients will be informed of any amendments that impact their participation in the trial. Changes that do not impact the conduct of the trial, such as minor protocol corrections or administrative changes, will not be reported to patients.

#### Confidentiality

Patient medical information will be kept strictly confidential and handled in accordance with Good Clinical Practice (GCP) guidelines and the Personal Health Information Protection Act (PHIPA). Each study patient will be given a unique identification number and all study case report forms will be kept in a locked filing cabinet in a secure office. We will not collect any personal identifiers that are not absolutely necessary for the success of this study. All data will be anonymized for data validation and analysis.

If a breach of personal health information (PHI) were to occur, all patients involved, the HiREB, and the McMaster Children’s Hospital Privacy Office will be notified. The study team will determine how the breach occurred and will put measures in place to ensure that another breach does not occur.

#### Progression criteria

Progression criteria will serve as important benchmarks to determine the feasibility of a larger RCT. While we take each criterion seriously, we recognize that feasibility is a multifaceted assessment and will not pause progression based solely on one factor. Progression criteria are detailed in Table 2.

**Table 2:** Progression criteria

Domain	Progression criteria
Recruitment and enrollment	Achieve a recruitment rate of at least 60% of eligible patients within the study period
Adherence to the intervention	Ensure that at least 70% of participants complete the prescribed pre-operative bowel preparation regimen
Follow-up and retention	Maintain a follow-up rate of at least 80% for post-operative assessments at 2 weeks and 4 weeks post-surgery
Logistics and resource utilization	Ensure the trial can be conducted within the allocated budget of $35,000 including personnel salaries and study-related expenses
Feasibility of randomization	Implement a randomization process with minimal delays and ensure allocation concealment to maintain the integrity of randomization
Feasibility of blinding	Determine if it is feasible to maintain blinding of surgeons, clinical outcome assessors, and data analysts throughout the study
Safety and adverse events	Monitor adverse events, aiming for no more than one severe adverse event related to the intervention per 30 participants

#### Dissemination

The results of the feasibility study will be published in a peer-reviewed journal and presented at relevant academic conferences. To enhance transparency and the quality of reporting, findings will be presented in accordance with the CONSORT 2010 extended statement for randomized pilot and feasibility trials [20].

## Discussion

Surgical wound infections are a major cause of morbidity in patients undergoing elective colorectal surgery, occurring in up to 25% of all cases [1]. The rate of wound infection is dependent on a myriad of factors including patient comorbidities, procedure performed, surgical technique and practice variability, prophylactic measures, and many other nuances that can vary from one patient to another.

 Research on pediatric wound infection rates after colorectal surgery is limited. Most of these studies are retrospective in nature, encompass overly heterogeneous groups, and consequently dilute results to insignificant outcomes.

Since the inception of the speciality, pediatric surgeons have been entrusted with producing high-quality research to inform best practices. Despite this, much of the evidence base in pediatric surgery is built upon retrospective studies [21, 22], with less than 2% of published literature utilizing RCTs [22, 23]. Evidently, there is a clear need for more rigorous study designs to improve the quality of evidence in pediatric surgery.

Given the paucity of evidence in pediatric literature, much of our current practice is extrapolated from studies consisting of predominantly adult populations. With no large pediatric RCTs, data available from retrospective studies have demonstrated mixed results. While the addition of OA and MBP appears to be beneficial in adult patients [7, 8], conflicting results are seen in children [9, 10]. Bearing in mind the disparity between pediatric and adult patients, further investigation is warranted.

To our knowledge, this is the first RCT to explore pre-operative MBP and prophylactic OA for pediatric patients undergoing elective colorectal surgery. Before conducting a fully powered, multicentre trial, it is imperative to carefully assess the research and intervention process [13]. The results of this study will provide important data on study design, recruitment and randomization, appropriate statistical endpoints, protocol deviations, logistical considerations, and risk assessment. Additionally, this study will be the first step in producing higher quality evidence—evaluating the utility of bowel preparation regimens for colorectal surgery in this patient population. As current bowl preparation practices are driven largely by surgeon preferences, the implications of this study could have a significant impact on clinical practice.

Upon study completion, we hope to conduct a fully powered, multicentre trial. To support these efforts, the study will be submitted to the Canadian Consortium of Research in Pediatric Surgery (CanCORPS). If accepted, all fifteen participating CanCORPS centers will be invited to participate. Ultimately, our goal is to determine the optimal pre-operative bowel regimen for pediatric patients undergoing elective colorectal surgery. Once this is identified, it can be incorporated into best practice guidelines to improve care for all pediatric patients undergoing these elective procedures.

## Data Availability

N/A.

## Abbreviations

ACS: American College of Surgeons
AE: Adverse event
APSA: American Pediatric Surgical Association
CI: Confidence interval
DSMB: Data Safety and Monitoring Board
GCP: Good Clinical Practice
HiREB: Hamilton Integrated Research Ethics Board
MBP: Mechanical bowel preparation
MPSRC: McMaster Pediatric Surgery Research Collaborative
MRN: Medical record number
NSQIP: National Surgical Quality Improvement Program
OA: Oral antibiotics
PHI: Personal health information
PHIPA: Personal Health Information Protection Act
RCT: Randomized controlled trial
REDCap: Research Electronic Data Capture
SAE: Serious adverse event
SSI: Surgical site infection
